# Effect of Hamstring Exercises on Morphological Parameters of the Hamstring Muscle: A Systematic Review With MRI and USI Findings

**DOI:** 10.1177/19417381261463404

**Published:** 2026-07-16

**Authors:** Gamze Arin-Bal, Merve Comlekci, Busra Pakoz, Ozgun Uysal, Volga Bayrakci-Tunay

**Affiliations:** † Department of Musculoskeletal Physiotherapy and Rehabilitation, Faculty of Physical Therapy and Rehabilitation, Hacettepe University, Ankara, Türkiye; ‡ Department of Sports Physiotherapy and Rehabilitation, Faculty of Physical Therapy and Rehabilitation, Hacettepe University, Ankara, Türkiye; § Department of Physiotherapy and Rehabilitation, Faculty of Health Sciences, I.zmir Katip Çelebi University, I.zmir, Türkiye

**Keywords:** exercise, hamstring muscles, magnetic resonance imaging, ultrasonography

## Abstract

**Context:**

Hamstring muscle strain is a common sports injury associated with significant time loss and high recurrence rates among athletes. Eccentric exercises, particularly the Nordic hamstring exercise (NHE), are effective in reducing injury incidence, yet their morphological adaptations remain underexplored.

**Objective:**

To examine exercise-induced morphological adaptations of the hamstring muscles and to provide an integrated perspective on changes in muscle architecture and size.

**Data Sources:**

In accordance with the Preferred Reporting Items for Systematic Reviews and Meta-Analyses (PRISMA) guidelines, PubMed, Scopus, Cochrane Library, and Web of Science were searched up to December 2024.

**Study Selection:**

Randomized controlled trials including adults (≥18 years old), with or without hamstring injuries, that investigated hamstring-strengthening interventions and assessed postintervention muscle morphology using magnetic resonance imaging (MRI) or ultrasonography were included.

**Study Design:**

Systematic review.

**Level of Evidence:**

Level 1.

**Data Extraction:**

Information on sample characteristics, exercise prescription, intervention details, exercise type, assessment device, targeted muscles, measured parameters, and outcomes was extracted.

**Results:**

A total of 14 studies, involving 382 participants, were included. Eccentric exercises demonstrated the most consistent adaptations, particularly increases in fascicle length (FL) and reductions in pennation angle (PA) in the biceps femoris long head (BFlh). Evidence for changes in muscle volume (MV) and cross-sectional area (CSA) was more heterogeneous across intervention types and imaging modalities. Muscle thickness showed limited or inconsistent changes across studies. Ultrasound (US) and MRI assessed different aspects of muscle morphology, limiting direct comparison across modalities. MRI-based studies more consistently demonstrated changes in MV and CSA, whereas US-based studies reported primarily architectural parameters such as FL and PA.

**Conclusion:**

Eccentric and resistance-based training were associated with favorable morphological adaptations in the hamstring muscles. Among these, eccentric strengthening interventions, particularly NHE-based protocols, were associated most consistently with increases in FL and reductions in PA of the BFlh. These findings highlight the importance of targeted exercise prescription and underscore the role of imaging modalities such as US and MRI in monitoring hamstring muscle adaptations.

Hamstring muscle strain is one of the most common injuries in sports, accounting for approximately 25% of time-loss injuries. It is associated with several well-established risk factors, including high sprinting exposure, eccentric muscle weakness, shorter fascicle length (FL), and a history of previous injury.^6,7,17,27^ Consequently, the prevention and rehabilitation of hamstring injuries have been investigated extensively over recent decades, aiming to reduce time lost from training and competition as well as the high recurrence rate, which has been reported to be as high as 30%.^12,13,25,26,28^ Researchers have examined various risk factors and investigated multiple strategies to reduce injury risk.^10,11,23,24^ Among the various preventive strategies investigated, eccentric training - particularly the Nordic hamstring exercise (NHE) - has shown the most consistent evidence for reducing hamstring injury incidence.^41^ Eccentric loading has also been incorporated into rehabilitation programs for hamstring strain injury, with emerging evidence supporting its early and progressive introduction.^14,43^

Studies have indicated that eccentric hamstring exercises reduce both the risk of hamstring injury and the rate of injury recurrence.^29,40,41^ In light of this substantial evidence, Union of European Football Associations (UEFA), and, consequently, many professional football clubs, implemented new evidence-based warm-up procedures aimed at reducing injury risk.^19^ Although these programs have led to a marked reduction in hamstring muscle strain incidence, hamstring strains remain among the most common sports injuries.^39,41^ Given that strength training is an inherent component of athletic practice, athletes are already exposed routinely to strengthening exercises. Therefore, the beneficial effects of the NHE may not be attributable solely to increases in muscle strength but may also be related to exercise-induced morphological adaptations. Various methods are available to quantify muscle morphology, with magnetic resonance imaging (MRI) and ultrasound (US) being the techniques used most commonly.^36^ MRI provides high-resolution images of muscle architecture and can capture physiological changes in muscle tissue after exercise.^16^ US, on the other hand, is more accessible and is used widely to measure muscle thickness (MT); however, assessing larger muscles often requires the use of panoramic US.^1,8^ This systematic review aims to investigate the effects of hamstring-strengthening exercises on the morphological characteristics of the hamstring muscles to provide a comprehensive understanding of exercise-induced adaptations.

## Methods

### Research Framework

**Figure 1.**
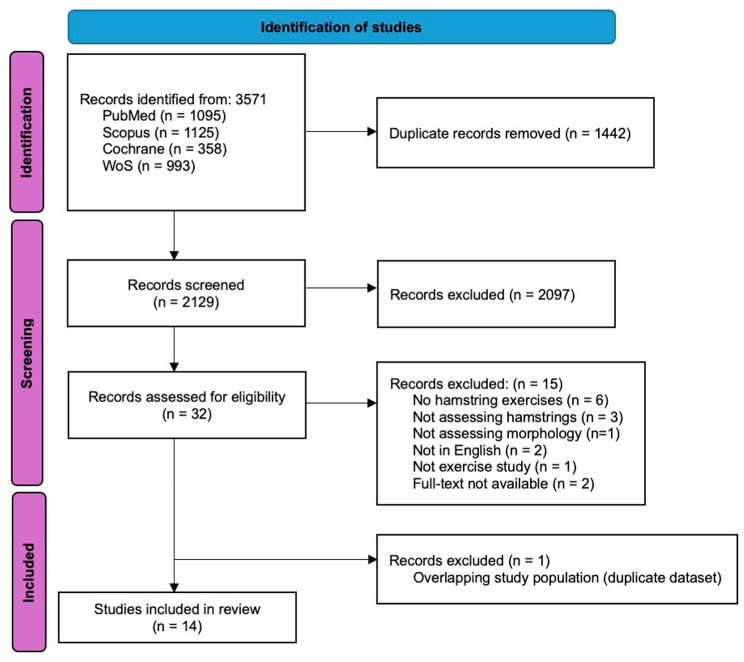
PRISMA flow chart of the systematic literature review. PRISMA, Preferred Reporting Items for Systematic Reviews and Meta-Analyses; WoS, Web of Science.

This review was conducted in accordance with the Preferred Reporting Items for Systematic Reviews and Meta-Analyses (PRISMA) guidelines. The study protocol was registered prospectively in the International Prospective Registry of Systematic Reviews (PROSPERO; registration number: CRD42024579964) on September 1, 2024, before the study selection process. Patients or public partners were not involved in the design, conduct, or interpretation of this systematic review. The study selection process is illustrated in the PRISMA flow diagram (Figure 1), in accordance with PRISMA guidelines.

### Literature Search Strategy

We conducted a systematic search of 4 databases - PubMed, Scopus, Cochrane Library, and Web of Science - from their inception to December 31, 2024. The search strategy was formulated using a combination of Medical Subject Headings (MeSH) terms and free-text keywords. In each database, an advanced search was conducted with the following string: ([hamstring OR “biceps femoris” OR semimembranosus OR semitendinosus] AND [exercise OR prevent* OR rehabilitation OR intervent*]) AND (“magnetic resonance imaging” OR “MRI” OR ultrasonography OR ultrasound OR ultrason*). One author imported all the identified studies to EndNote and Rayyan systematic review software, where duplicates were removed using both software.

### Study Selection

After a comprehensive search, the titles and abstracts of each study were screened using the Rayyan software and classified as “include,” “exclude,” or “maybe” by 2 independent, blinded researchers. Any conflicts were resolved by another 2 reviewers. Eligibility assessment was carried out based on the inclusion criteria. Full texts of studies that met the inclusion criteria were retrieved. Finally, detailed documentation was prepared for all included studies, covering information about participants, interventions, outcome measures, and study design.

A formal assessment of clinical heterogeneity was performed by comparing systematically study populations, intervention characteristics, outcome measures, and imaging modalities. Given the substantial heterogeneity in study designs, intervention protocols, and morphological assessment methods, a quantitative meta-analysis was deemed inappropriate. Therefore, the findings were synthesized using a narrative approach.

### Eligibility Criteria

Studies were eligible for inclusion if they met the following criteria: (1) randomized controlled trials (RCTs) investigating exercise interventions specifically designed to strengthen the hamstring muscles (e.g., eccentric, concentric, resistance-based, or combined strengthening programs); (2) assessment of hamstring muscle morphology; (3) evaluation of hamstring muscle morphology using MRI and/or ultrasonography; (4) participants aged ≥18 years, including healthy people or people with hamstring injury; (5) full-text articles published in English. Studies were excluded if they investigated interventions not intended primarily to strengthen the hamstring muscles (e.g., stretching-only protocols), or if they were review articles, conference abstracts, case reports, case series, observational studies, nonrandomized trials, or studies involving participants with conditions other than hamstring injury or with a history of surgery.

### Data Extraction

Data extraction was conducted independently by 2 physiotherapists, both PhD candidates with experience in sports and musculoskeletal physiotherapy research. Standardized data extraction tables were used to collect information on study characteristics (e.g., author and year), participant characteristics (e.g., age, sex, and population), intervention details (e.g., exercise type and duration), assessment tools, outcome measures, and key findings. In cases of disagreement, the 2 reviewers first attempted to reach consensus through discussion. If consensus could not be achieved, a third reviewer - a physiotherapist with a PhD - was consulted. When necessary, a fourth reviewer, also a physiotherapist with a PhD, provided additional support to resolve any remaining disagreements.

### Quality of Assessment

Methodological quality was assessed using the PEDro scale, and risk of bias was evaluated using the Cochrane Collaboration’s risk of bias tool. PEDro scores were categorized as high (7 to 10), moderate (4 to 6), or low (≤3). For the Cochrane risk of bias assessment, each RCT was evaluated across the domains of selection bias, performance bias, detection bias, attrition bias, reporting bias, and other sources of bias, with judgments categorized as low, high, or unclear risk of bias. In addition, the included studies were appraised using the EBRO (Dutch Evidence-based Richtlijn Ontwikkeling) checklist, and levels of evidence were assigned accordingly. Quality assessment was conducted independently by the same reviewers involved in data extraction, and any disagreements were resolved as described in “Data Extraction.”

## Results

### Study Selection and Quality Assessment

The initial search yielded 3571 records. After duplicate removal, 2129 articles were screened for eligibility. After full-text assessment, 15 studies were considered eligible for inclusion. However, 2 articles were identified as reporting results from the same dataset based on identical participant characteristics, author group, and methodology. To avoid double counting, only the earlier publication was retained,^23^ and the later publication was excluded.^42^ Consequently, 14 unique studies were included in the final review. The study selection process is illustrated in the PRISMA flow diagram, which details the reasons for exclusion at the screening and full-text eligibility stages along with the number of records in each category (Figure 1).

The following information was extracted from each study: first author, publication year, population details, exercise protocol details, exercise type, assessment device, measured parameters, muscles assessed, and main findings (Table 1). The mean methodological quality score was 6.6 ± 0.9 points according to the PEDro scale. Overall, the included studies were of moderate to high methodological quality, with PEDro scores ranging from 5 to 8 out of 10 (Table 2). Most studies (n = 9) demonstrated high methodological quality, achieving scores of ≥7, while 5 studies were rated as moderate quality with scores of 5 or 6. According to the EBRO checklist, all studies were rated as level B evidence.

**Table 1.** Descriptives of the selected studies

Authors (year)	Population	Descriptives, n (or group); age; weight; height	Exercise prescription	Intervention type	Exercises	Device	Parameters	Muscles	Results
Ruas et al^35^ (2018)	Young men: CON/CON, n = 10; ECC/ECC, n = 10; CON/ECC, n = 10; no training, n = 10	n = 40; 22.9 ± 2.3 years; 70.7 ± 11.0 kg; 174.3 ± 6.9 cm	2 out of 7 days for 6 weeks of training for 20 mins each	IG, concentric and eccentric and combined training on an isokinetic dynamometer; CG, none	IG, hamstrings concentric (CON/CON) or eccentric (ECC/ECC) or combination (CON/ECC) muscle action training protocols; CG, no interventions	US; B-mode; linear-array transducer; 4.2-10.3 MHz	MT	BFlh, ST, SM	All training groups increased hamstring MT. Combined and ECC protocols produced greater adaptations than the CON protocol, whereas no changes were observed in the CG
Bradley et al^2^ (2023)	Healthy recreationally active: IG, n = 9;M/F: 6/3; CG, n = 11; M/F:4/7	n = 20; 30.2 ± 7.7 years; 69.6 ± 15.0 kg; 166.8 ± 7.3 cm	2 out of 7 days for 4 weeks	IG, concentric training and BFR; CG. rowing and lifting	IG, concentric training (rowing at 40% maximum power output and lifting 30% 1-RM for 1 × 20 reps and 3 × 10 reps) and BFR (applied to thighs bilaterally at 80% occlusion); CG, rowing at 80% power and lifting 60% 1-RM for 1 × 10 reps and 3 × 5 reps	US, 4C-RS convex transducer, 12 MHz	CSA	BF	CSA improved after the intervention; however, no significant between-group differences were observed
Marušicˇ et al^23^ (2020)	Healthy and injury-free volunteers: IG, n = 18, M/F: 12/6; CG, n = 16, M/F: 12/4	IG: 24.2 ± 2.1 years, 73.0 ± 14.3 kg, 177 ± 9 cm; CG, 23.0 ± 2.8 years, 75.1 ± 15.1 kg, 179 ± 7 cm	2 out of 7 days for 6 weeks	IG, progressive eccentric training (modified NHE and glider); CG, continuing regular recreational habits	IG, 2 ECC hamstring exercises in a lengthened position: the modified NHE and the glider; CG, no intervention	US, linear probe	MT, PA, FL	BFlh	ECC training decreased PA and increased BFlh FL
Ribeiro-Alvares et al^34^ (2018)	Healthy young adults: IG, n = 10, M/F: 3/7; CG, n = 10,M/F: 3/7	IG, 23.7 ± 3.3 years, 59.1 ± 12.8 kg 165.1 ± 9 cm; CG, 26.0 ± 2.7 years 63.7 ± 11.1 kg, 166.4 ± 7.2 cm	2 out of 7 days for 4 weeks	IG, NHE; CG, none	IG, NHE, 3 sets of 6 to 10 repetitions; CG, no intervention	US, B-mode, 4 cm linear array probe, 13 MHz	MT, PA, FL	BFlh	NHE did not change MT but resulted in a greater decrease in PA and a greater increase in BFlh FL compared with the CG
Frouin et al^9^ (2024)	Recreationally active participants: IG1, n = 11, M/F: 6/5; IG2, n = 13, M/F: 9/4; CG, n = 12, M/F: 8/4	IG1, 21.6 ± 3.1 years, 69.4 ± 11 kg, 174.9 ± 8.8 cm; IG2, 21.6 ± 2.9 years, 68.0 ± 8.9 kg, 175.0 ± 8 cm; CG, 23.4 ± 4 years, 68.4 ± 8.4 kg, 174.2 ± 10.9 cm	3 out of 7 days for 9 weeks	IG1, high-load resistance training program; IG2, low load resistance training program combined with BFR; CG, regular sports activities	Two training sessions (a and b) that used different exercises were alternated over the duration of the training program. Session (a), stiff-leg deadlift and front squat; session (b), bi-set of bilateral seated leg curl and seated leg extension. Each session was started by a 10-minute standardized warm-up	US, B-mode, 10-2 linear transducer and 20-6 linear transducer, 120 MHz	MV	BFlh, ST, SM	High-load resistance training produced greater increases in ST MV than the CG, whereas low-load resistance training combined with BFR produced greater increases in SM MV. No significant between-group differences were observed for biceps femoris MV
Manimmanakorn et al^22^ (2013)	Female netballers: IG1, n = 10; IG2, n = 10; CG, n = 10	n = 30, 20.2 ± 3.3 years, 65.2 ± 6.5 kg, 168.4 ± 5.8 cm	3 out of 7 days for 5 weeks	IG1, concentric training and hypoxic training; IG2, concentric training and BFR; CG, concentric training	Training: bilateral knee extensions and flexions. Each training session consisted of 3 sets of knee extensions followed by 3 sets of knee flexions to failure (total of 6 sets); IG1, training + BFR; IG2, training + hypoxicator; CG; training	MRI, 1.5T, coronal STIR images	CSA	Knee extensors and flexors as groups	Flexor muscle CSA increased in all groups, with greater increases observed in the BFR and hypoxic training groups than in the CG
Shamsehkohan et al^38^ (2012)	Athletes (hamstring with strain injury [grade I or II]): IG1, n = 8; IG2, n = 8; CG, n = 5	IG1, 27.5 ± 5.61 years, 68.5 ± 7.18 kg, 175 ± 7.8 cm; IG2, 24.62 ± 5.4 years, 71.12 ± 10.25 kg, 176 ± 6.62 cm; CG, 24.6 ± 3.21 years, 76.60 ± 12.05 kg, 178 ± 4.57 cm	4 out of 7 days for 4 weeks	IG1, HamSprint program; IG2, ECC exercises; CG, none	IG1, running, marching, skips, short stride cariocas, side shuffles, leg cycling, leg pawing, ankle pops, quick support running, forward falling running and explosive starts; IG2, ECC box drops, ECC loaded lunge drops, ECC forward pulls, split-stance Zerchers and single leg dead lift; CG, no intervention	MRI, 1.5T, spin-echo images	MV	Hams	Postintervention injured MV differed significantly between groups, with the HamSprint program showing a significant advantage over the CG. No significant difference was observed between the ECC and HamSprint groups
Vissing et al^44^ (2008)	Untrained healthy men: IG1, n = 8; IG2, n = 7	n = 15, 25.1 ± 3.9 years, 80.6 ± 16 kg, 181.3 ± 5.2 cm	3 out of 7 days for 12 weeks	IG1, conventional resistance training; IG2, plyometric training	IG1, leg press, knee extensions, and hamstring curl; IG2, countermovement jumps, hurdle jumps, and drop jumps	MRI, 1.5T, spin echo images	CSA	ST, SM, BF	CSA increased in all hamstring muscle groups after training, with no significant differences between the resistance and plyometric training groups
Lazarczuk et al^18^ (2024)	Recreationally active men: IG1, n = 10; IG2, n = 10;CG, n = 10	IG1, 21.6 ± 3.2 years, 85.0 ± 10.9 kg, 182.8 ± 8.7 cm; IG2, 23.1 ± 4.1 years, 81.6 ± 9.8 kg, 179.8 ± 1.6 cm; CG, 21.3 ± 3.7 years, 75.9 ± 11.8 kg, 178.5 ± 5.4 cm	2 out of 7 days a week for 10 weeks	IG1, NHE; IG2, hip extension, CG, none	IG1 and IG2, progressively overloaded: first week 2 × 6, second week 3 × 6, third week 4 × 7, fourth week 4 × 10, fifth to eighth weeks 5 × 8 to 10, ninth week 6 × 6, tenth week 5 × 5; CG, no intervention	MRI, 3T	MV, CSA	Hams	Muscle-tendon adaptations were region specific. The NHE group showed a greater increase in SM free tendon volume than the CG, whereas the hip extension group showed a greater increase in the BFlh MV-to-interface area ratio. Muscle CSA increased primarily in the midportion of the ST in both training groups and in the midportion of the BFlh in the hip extension group
Maeo et al^20^ (2024)	Healthy young men: IG1, n = 14; IG2, n = 14; CG, n = 14	IG1, 25 ± 4 years, 77 ± 11 kg, 178 ± 6 cm; IG2, 27 ± 3 years, 76 ± 13 kg, 176 ± 8 cm; CG, 24 ± 3 years, 73 ± 6 kg, 178 ± 7 cm	3 out of 7 days a week for 12 weeks; 34 supervised sessions over 12 weeks (3 times per week apart from weeks 1 and 12 (2 times per week)	IG1, NHE; IG2, lengthened state ECC training; CG, none	IG1 and IG2, systematic, progressive knee flexor training of both legs. All training sessions began with a standardized cycling warm-up. This was followed by 2× ~15 seconds of static stretches of the hamstrings of each leg in a standing position; CG, no intervention	MRI, 3T	MV	BFlh, BFsh, ST, SM	Both ECC training protocols increased hamstring MV, although the hypertrophic response was exercise specific. The NHE group showed increases in most hamstring muscles except the SM, whereas the lengthened-state ECC training group showed increases in all constituent hamstring muscles. No changes were observed in the CG
Carmichael et al^3^ (2022)	Recreationally active men; IG1, n = 12; IG2, n = 12	IG1, 21.7 ± 4.9 years, 73.0 ± 9.4 kg, 177.1 ± 5.0 cm; IG2, 22.0 ± 3.6 years; 77.8 ± 11.1 kg; 181.9 ± 6.2 cm	2 out of 7 days for 6 weeks, followed by 4 weeks of detraining	IG1, ECC hip extension exercise intervention; IG2, isometric hip extension exercise intervention	Unilateral hip extension exercise performed with either eccentric or isometric contraction mode	US, 2-dimensional US + MRI	FL, MV	BFlh, SM, ST	The ECC hip extension intervention significantly increased BFlh FL (+19.7%) and increased BFlh (+13.3%) and SM MV (+12.5%). The isometric hip extension intervention increased strength and was associated with selective ST hypertrophy, with no significant increase in BFlh FL
Pollard et al^31^ (2019)	Recreationally active men; IG1, n = 10; IG2, n = 10; IG3, n = 10	n = 30, 24 ± 4 years, 78 ± 11 kg, 181 ± 6 cm	128 total repetitions across 6 weeks, followed by 4 weeks of detraining	IG1, NHE bodyweight; IG2, NHE weighted; IG3, RHC weighted	NHE (bodyweight), NHE (weighted), RHC (weighted)	US	FL	BFlh	The weighted NHE group showed the greatest increase in BFlh FL (+1.57 cm). The bodyweight NHE group showed moderate architectural adaptation, whereas the weighted RHC group showed negligible change in BFlh FL. FL declined after detraining, especially in the weighted NHE group
Presland et al^33^ (2018)	Recreationally active men: IG1, n = 10; IG2, n = 10	n = 20, 22.3 ± 2.8 years, 75.1 ± 8.8 kg, 179.1 ± 7.7 cm	Standardized NHE training for 2 weeks, followed by 4 weeks of either high- or low-volume NHE training, total intervention duration: 6 weeks, followed by 4 weeks of detraining	IG1, high-volume ECC NHE training; IG2, low-volume ECC NHE training	IG1, high-volume ECC NHE training; IG2, low-volume eccentric NHE training	US	FL	BFlh	Both high- and low-volume NHE training significantly increased BFlh FL after 6 weeks (high volume: +23%; low volume: +24%). These architectural adaptations declined after 2 weeks of detraining
Presland et al^32^ (2020)	Healthy men: IG1, n = 10; IG2, n = 10	n = 20, 27.8 ± 5.3 years, 80.0 ± 10.7 kg, 178.4 ± 7.7 cm	6 weeks of inertial flywheel resistance training, followed by 4 weeks of detraining	IG1, conventional flywheel leg-curl training; IG2, eccentrically biased flywheel leg-curl training	Inertial flywheel leg-curl training; conventional vs eccentrically biased prescription	US	FL	BFlh	The eccentrically biased flywheel intervention significantly increased BFlh FL (+14 ± 5%), whereas the conventional flywheel intervention did not change FL. The architectural gains declined after detraining

1-RM, 1-repetition maximum; BF, biceps femoris; BFlh, biceps femoris long head; BFR, blood flow restriction; BFsh, biceps femoris short head; CG, control group; CON, concentric; CSA, cross-sectional area; ECC, eccentric; F, female; FL, fascicle length; IG, intervention group; M, male; MRI, magnetic resonance imaging; MT, muscle thickness; MV, muscle volume; PA, pennation angle; reps, repetitions; RHC, razor hamstring curl; SM, semimembranosus; ST, semitendinosus; STIR, short-tau inversion recovery; US, ultrasound imaging.

**Table 2.** Results of methodologic quality assessment via PEDro scale

Article (year)	Eligibility criteria	Random allocation	Concealed allocation	Similarity of groups at baseline	Blind subject	Blind therapist	Blind assessor	Adequate follow-up	Intention to treat analysis	Between group analysis	Point estimates and variability	Total score
Ruas et al^35^ (2018)	●	●	○	○	○	○	○	●	●	●	●	5
Bradley et al^2^ (2023)	●	●	○	●	○	○	●	●	●	●	●	7
Marušicˇ et al^23^ (2020)	●	●	○	●	○	○	●	●	●	●	●	7
Ribeiro-Alvares et al^34^ (2018)	●	●	●	●	○	○	●	●	●	●	●	7
Presland et al^33^ (2018)	●	●	○	●	○	○	●	●	○	●	●	6
Presland et al^32^ (2020)	●	●	○	●	○	○	●	●	●	●	●	7
Pollard et al^31^ (2019)	●	●	○	●	○	○	●	●	●	●	●	7
Carmichael et al^3^ (2022)	●	●	○	●	○	○	●	●	○	●	●	6
Frouin et al^9^ (2024)	●	●	○	●	●	●	●	●	○	●	●	8
Manimmanakorn et al^22^ (2013)	○	○	○	●	○	○	●	●	●	●	●	6
Shamsehkohan et al^38^ (2012)	●	●	○	●	○	○	○	●	○	●	●	5
Vissing et al^44^ (2008)	●	●	○	●	○	○	●	●	●	●	●	7
Lazarczuk et al^18^ (2024)	●	●	○	●	○	○	●	●	●	●	●	7
Maeo et al^20^ (2024)	○	●	○	●	○	○	●	●	●	●	●	7
Number of studies meeting criterion	13	14	1	14	1	1	13	16	12	16	16	

●, criteria met; ○, criteria not met.

### Study Designs

All included studies were RCTs. The intervention durations ranged from 4 weeks to 12 weeks. The included studies investigated a range of hamstring-strengthening exercise protocols, including resistance training, eccentric loading, blood flow restriction (BFR), hypoxic training, isometric training, and hybrid strengthening programs. When present, control groups either received no intervention or performed alternative exercise protocols not designed specifically to strengthen the hamstrings. Outcome measures were assessed consistently before and after intervention periods.

### Devices

Among the included studies, muscle morphology was assessed primarily using 2 imaging modalities: US and MRI. Conventional B-mode US was the modality used most frequently and was employed mainly to assess architectural parameters such as FA, pennation angle (PA), and MT. MRI was used to evaluate muscle volume (MV) and cross-sectional area (CSA), either as the sole imaging method or in combination with US in some studies. Device specifications were not reported consistently across studies, although several studies described the use of linear-array transducers with frequencies ranging from 7.5 MHz to 12 MHz for US assessments. Despite some variation in imaging protocols, measurements were typically obtained at standardized anatomical locations, such as the mid-thigh or two-thirds of femur length, to evaluate hamstring morphology.

### Population

The participant characteristics of the included studies are summarized in Table 1. The 14 included studies involved 382 participants in total, with sample sizes ranging from 15 to 42 participants per study. The majority of studies recruited male participants, primarily young adults. Across the included studies, a total of 284 male and 77 female participants were reported, whereas 1 study did not report participant sex.^38^ One study involved exclusively female participants.^22^ Participants were generally healthy, with most studies involving recreationally active, physically active, or untrained people; only 1 study included athletes with hamstring strain injury.^38^ Inclusion criteria typically required no recent history of lower limb injury or resistance training within the previous 6 months. Exclusion criteria commonly included neuromuscular disorders, recent surgery, or inconsistent training adherence. The mean age of participants across the studies ranged from 20.2 ± 3.3 to 30.2 ± 7.7 years. Across the included studies, the reported mean weight ranged approximately from 59.1 kg to 85.0 kg, and mean height ranged from 1.65 m to 1.83 m.

### Effects of Interventions on Hamstring Muscle Morphology

#### Concentric and Resistance-Based Interventions

Several studies examined concentric or resistance-based exercise interventions. Ruas et al^35^ reported significant increases in MT of the semitendinosus (ST) and semimembranosus (SM) muscles after resistance training. Frouin et al^9^ demonstrated increases in MV across different hamstring muscle heads in both intervention groups, although hypertrophy patterns varied between muscles. In participants with hamstring injury, Shamsehkohan et al^38^ reported differences in postintervention injured MV between groups after eccentric-based rehabilitation and HamSprint exercise programs. Bradley et al^2^ and Manimmanakorn et al^22^ reported increases in CSA after resistance-based or BFR interventions; however, these changes were not significantly greater than those observed in active control groups when assessed using US or MRI. Similarly, Vissing et al^44^ reported increases in CSA after training, although no nonexercising control group was included. Carmichael et al^3^ showed that an isometric hip extension intervention was associated with selective ST hypertrophy but did not result in significant increases in biceps femoris long head (BFlh) FL. Likewise, Presland et al^32^ reported that conventional flywheel leg-curl training did not alter BFlh FL, suggesting that resistance training alone may be insufficient to induce consistent architectural adaptation. Overall, concentric and resistance-based interventions appeared to contribute more consistently to hypertrophic changes such as MT, MV, and CSA, whereas their effects on FL and PA were less evident.

#### Eccentric-Based Interventions

Eccentric exercise interventions, particularly NHE-based protocols, were investigated most frequently. Ruas et al^35^ reported increases in MT after eccentric loading. Marušicˇ et al^23^ and Ribeiro-Alvares et al^34^ demonstrated significant increases in BFlh FL, accompanied by decreases in PA, after eccentric training performed at long muscle lengths or using NHE-based protocols. Ribeiro-Alvares et al^34^ reported no significant changes in MT, which may be related to the lower training volume and shorter intervention duration compared with other eccentric protocols. Additional eccentric-focused studies further supported these findings. Pollard et al^31^ showed that weighted NHE produced greater increases in BFlh FL than bodyweight NHE, whereas the razor hamstring curl induced comparatively limited architectural adaptation. Presland et al^33^ also found that both high- and low-volume NHE training increased BFlh FL significantly after 6 weeks, indicating that eccentric NHE-based loading can induce meaningful architectural adaptation even at lower training volumes. Carmichael et al^3^ demonstrated that eccentric hip extension training significantly increased BFlh FL and was accompanied by increases in BFlh and SM MV. Similarly, Presland et al^32^ reported that eccentrically biased flywheel leg-curl training significantly increased BFlh FL, whereas a conventional flywheel approach did not. MRI-based studies by Lazarczuk et al^18^ and Maeo et al^20^ further demonstrated increases in MV and region-specific CSA after eccentric or lengthened-state training compared with control conditions.

Overall, the muscles evaluated most frequently across interventions included the BFlh, ST, and SM. Across the included studies, eccentric-based strengthening interventions demonstrated the most consistent adaptations in FL and PA, particularly in the BFlh, whereas evidence for changes in MV and CSA was more heterogeneous across intervention types and imaging modalities.

### Quality of Assessment

Only 1 study was rated as having a low risk of both performance bias and selection bias according to the Cochrane Collaboration's Tool (Table 3).^9^ Seven studies (50.0%) were rated as having an unclear risk of bias for random sequence generation, and 11 studies (78.6%) were rated as having an unclear risk of bias for allocation concealment. All studies except Frouin et al^9^ were assessed as having a high risk of performance bias because of the lack of blinding of participants and researchers. For detection bias, 11 studies were rated as low risk, 2 as high risk, and 1 as unclear. A total of 12 studies were assessed as having a low risk of attrition bias, whereas 2 studies were rated as unclear. All included RCTs were assessed as having a low risk of bias for both selective reporting and other sources of bias.

**Table 3.** Risk of bias assessment of included RCT using Cochrane Collaboration’s tool

Article (year)	Random sequence generation (selection bias)	Allocation concealment (selection bias)	Blinding of participants and researchers (performance bias)	Blinding of outcome assessment (detection bias)	Incomplete outcome data (attrition data)	Selective reporting (reporting data)	Other bias
Ruas et al^35^ (2018)	●	●	●	●	●	●	●
Bradley et al^2^ (2023)	●	●	●	●	●	●	●
Marušicˇ et al^23^ (2020)	●	●	●	●	●	●	●
Ribeiro-Alvares et al^34^ 2018	●	●	●	●	●	●	●
Presland et al^33^ (2018)	●	●	●	●	●	●	●
Presland et al^32^ (2020)	●	●	●	●	●	●	●
Pollard et al^31^ (2019)	●	●	●	●	●	●	●
Carmichael et al^3^ (2022)	●	●	●	●	●	●	●
Frouin et al^9^ (2024)	●	●	●	●	●	●	●
Manimmanakorn et al^22^ (2013)	●	●	●	●	●	●	●
Shamsehkohan et al^38^ (2012)	●	●	●	●	●	●	●
Vissing et al^44^ (2008)	●	●	●	●	●	●	●
Lazarczuk et al^18^ (2024)	●	●	●	●	●	●	●
Maeo et al^20^ (2024)	●	●	●	●	●	●	●

●, low risk of bias; ●, unclear risk of bias; ●, high risk of bias; RCT, randomized controlled trial.

## Discussion

This systematic review aimed to evaluate the effects of hamstring-strengthening exercises on muscle morphology outcomes assessed by US and MRI. Across the included studies, eccentric and resistance-based interventions were associated with changes in FL, MV, and CSA, and, to a lesser extent, PA and MT. However, the consistency of these adaptations varied according to intervention type, muscle assessed, and imaging modality. Overall, the most consistent finding was an increase in FL after eccentric strengthening interventions, particularly in the BFlh.

Eccentric strengthening interventions, particularly NHE-based protocols, were the most consistently associated with favorable architectural adaptations in the hamstring muscles. Acute responses to NHE have been shown to include increases in MT and PA of the BFlh and ST, although these changes appear to return to baseline within a short period.^21^ Over longer intervention periods, both NHE and Romanian deadlift-based training have been associated with increased FL and decreased PA across the hamstrings,^5^ with elongation of the BFlh fascicles linked to enhanced muscle function and a reduced risk of strain injury.^5,23^ In addition, NHE has demonstrated the ability to increase hamstring CSA, and this effect may persist even after a period of detraining.^5^ NHE training over 6 weeks has also been associated with gains in MV and CSA, particularly in the SM, although FL did not change in any study.^18,37^ These findings are further supported by additional evidence indicating that weighted NHE may produce greater increases in BFlh FL than bodyweight NHE, whereas the razor hamstring curl appears to induce comparatively limited architectural adaptation.^31^ Similarly, both high- and low-volume NHE training have been associated with increased BFlh FL after 6 weeks, suggesting that eccentric NHE-based loading can promote meaningful architectural adaptation even at lower training volumes.^33^ Taken together, these findings suggest that eccentric interventions produce the most consistent architectural adaptations, especially in BFlh FL and PA.

MRI-based studies also supported the role of eccentric or lengthened-state strengthening in promoting hypertrophic adaptations. Increases in MV and region-specific CSA after eccentric or lengthened-state training were observed in several studies,^18,20^ and similar findings have also been reported for eccentric hip extension interventions, which were associated with increased BFlh FL together with increases in BFlh and SM MV, whereas isometric training appeared to produce a different and more selective adaptation pattern.^3^ However, these changes were not uniform across all hamstring muscles, suggesting that adaptation patterns may depend on exercise selection, loading characteristics, contraction mode, and the specific architectural or morphological parameter being assessed. This is also consistent with the idea that different eccentric exercises may target different regions or heads of the hamstring muscle group preferentially.

Resistance-based and combined strengthening interventions were also associated with morphological changes, although findings were less consistent than those observed for eccentric training. Ruas et al^35^ reported increases in MT after concentric, eccentric, and combined muscle action training. Frouin et al^9^ demonstrated increases in MV in different hamstring heads after both high-load resistance training and low-load resistance training combined with BFR, although the hypertrophy pattern differed across muscles. Similarly, Bradley et al^2^ and Manimmanakorn et al^22^ reported increases in CSA after resistance-based or BFR-related interventions, but these changes were not clearly superior to those observed in active control conditions. Vissing et al^44^ also observed increases in CSA after training, although the absence of a nonexercising control group limits the interpretation of those findings. Notably, none of the concentric or resistance-based studies included in this review evaluated FL directly, which limits comparison with eccentric training studies in terms of architectural adaptation. This interpretation is further supported by evidence from flywheel leg-curl training, in which a conventional resistance-based approach did not alter BFlh FL, whereas an eccentrically biased prescription resulted in significant fascicle lengthening.^32^ Taken together, these findings suggest that eccentric bias, rather than resistance training alone, may be a key determinant of architectural adaptation.

Low-load BFR training may stimulate muscle adaptation without high mechanical loading because of increased metabolic stress and hypoxic conditions in the muscle. In this review, 3 studies investigated the effects of BFR-related interventions on hamstring morphology. One study reported no effect on MV,^9^ which is in line with previous literature,^30,45^ whereas 2 others demonstrated increases in CSA.^2,22^ These inconsistent findings may reflect differences in occlusion pressure, exercise type and intensity, intervention duration, participant characteristics, and the specific muscles assessed. Therefore, the current evidence suggests that the effectiveness of BFR in inducing morphological adaptations in the hamstrings is context-dependent and less consistent than that of eccentric strengthening interventions.

From a clinical perspective, the observed increases in FL and changes in PA may be particularly relevant, as these adaptations have been linked to a lower risk of hamstring strain injury.^4,5,23^ Longer fascicles may improve the muscle’s capacity to tolerate high mechanical loads during sprinting and other high-speed activities, thereby contributing to injury resilience.^15^ This interpretation is further supported by additional studies reporting BFlh fascicle lengthening primarily after interventions with greater eccentric demand, while also showing that architectural gains may decline after periods of detraining in some protocols.^31-33^ This suggests not only that eccentric loading is important for stimulating favorable adaptations, but also that continued exposure may be necessary to maintain them. In contrast, resistance-based interventions appear to contribute more consistently to hypertrophic changes such as increases in MV or CSA, while their effects on FL remain less clear because this parameter was not assessed directly in several studies. Accordingly, the findings of this review suggest that different hamstring-strengthening interventions may produce distinct but potentially complementary morphological adaptations.

Despite these findings, the available evidence does not support a definitive conclusion that one strengthening intervention is superior to all others. Considerable heterogeneity existed across the included studies in terms of participant characteristics, training status, intervention type, exercise dosage, target muscles, outcome variables, and imaging methods. This heterogeneity limited direct comparison between studies and precluded a meaningful meta-analysis. Nevertheless, the narrative synthesis indicates that eccentric strengthening interventions, particularly NHE-based protocols, were associated most consistently with favorable architectural adaptations in the hamstrings, especially increased FL and decreased PA in the BFlh. The additional identified studies further support this pattern, particularly by suggesting that weighted NHE, eccentric hip extension, and eccentrically biased flywheel training may produce stronger fascicle-length responses than less eccentrically demanding alternatives.

The findings of this review highlight the relevance of targeted exercise prescription in both performance and rehabilitation settings. Clinicians and rehabilitation professionals may use these findings to select interventions according to the desired morphological adaptation. Interventions aimed at increasing FL and modifying muscle architecture may be particularly relevant for athletes at risk of hamstring strain injury, whereas resistance-based approaches may be useful when hypertrophic adaptations are also a goal. Future studies should use more standardized intervention protocols and imaging procedures, include more diverse samples, and compare strengthening interventions more directly to clarify which exercise characteristics are most strongly associated with specific morphological outcomes.

### Limitations

Despite the structured approach of this systematic review, several limitations must be acknowledged. First, substantial heterogeneity existed across the included studies in terms of participant characteristics, exercise protocols, target muscles, outcome measures, and imaging methods, which limited direct comparison and precluded meta-analysis. In addition, the use of different imaging modalities, including B-mode US, panoramic US, and MRI, may have influenced the consistency of reported morphological parameters because these methods capture different aspects of muscle morphology and rely on different measurement procedures. Although MRI is often considered the reference standard for assessing muscle CSA and volume due to its higher spatial resolution and lower operator dependency, US-based measurements may be more vulnerable to methodological variability. Furthermore, most studies included involved relatively small sample sizes and predominantly young, healthy male participants, which limits the generalizability of the findings to women, older adults, and clinical populations. Finally, although all included studies were RCTs, methodological quality varied across studies, and several were limited by lack of blinding and other sources of bias.

## Conclusion

Eccentric and resistance-based interventions were associated with favorable morphological adaptations in the hamstring muscles, particularly changes in FL, MV, and CSA. Among these, eccentric strengthening interventions, especially NHE-based protocols, were associated most consistently with increases in FL and reductions in PA of the BFlh. However, evidence for changes in MV and CSA was more heterogeneous across studies and imaging modalities. These findings highlight the importance of targeted exercise prescription when specific morphological adaptations are desired. Greater standardization of intervention protocols and assessment methods, together with the inclusion of more diverse participant groups, may improve the comparability and external validity of future research. Overall, this review underscores the role of imaging modalities such as US and MRI in monitoring hamstring muscle adaptations after strengthening interventions.
